# Oncological effectiveness of bladder-preserving trimodal therapy versus radical cystectomy for the treatment of muscle-invasive bladder cancer: a system review and meta-analysis

**DOI:** 10.1186/s12957-023-03161-z

**Published:** 2023-08-29

**Authors:** Xiaozhe Su, Caitao Dong, Wenbiao Liao, Wentao Liu

**Affiliations:** 1 Department of Urology, The Second Xiangya Hospital of Central South University, Changsha, China; 2 Department of Urology, Renmin Hospital of Wuhan University, Wuhan, China

**Keywords:** Muscle-invasive bladder cancer, Trimodal therapy, Radical cystectomy, Meta-analysis

## Abstract

**Objective:**

Radical cystectomy (RC) is the gold standard treatment for muscle-invasive bladder cancer (MIBC). As a bladder-preservation option recommended in guidelines, trimodal therapy (TMT) has become increasingly popular in recent years. However, it is still uncertain whether TMT can provide comparable oncologic outcomes to RC. Therefore, it is imperative to evaluate whether TMT yields comparable outcomes to RC.

**Methods:**

We conducted a systematic search of Web of Science, MEDLINE, the Cochrane Library, and EMBASE databases up to June 2023 to identify studies that met our inclusion criteria. The primary outcome measures evaluated in this study were overall survival (OS) and cancer-specific survival (CSS). The study quality was evaluated independently by two authors, and data were extracted accordingly.

**Results:**

After excluding duplicates and ineligible articles, our meta-analysis included seven studies involving 3,489 and 13,877 patients in the TMT and RC groups, respectively. Short-term overall survival rates were comparable between the groups, but beyond 5 and > 10-years, the RC group had significantly higher overall survival rates compared to the TMT group. In terms of cancer-specific survival, there was no significant difference between the groups at 1-year follow-up, but from the second year onwards, including the 5-year and > 10-year nodes, the RC group had significantly better outcomes compared to the TMT group.

**Conclusion:**

The treatment effect of RC is better than that of TMT. Unless the patient can’t tolerate RC or has a strong desire to preserve the bladder, RC should be chosen over TMT in treatment, and patients undergoing TMT should be closely followed up.

**Supplementary Information:**

The online version contains supplementary material available at 10.1186/s12957-023-03161-z.

## Introduction

Bladder cancer is a highly prevalent and lethal tumor of the urinary system [1, 2]. For patients with non-metastatic muscle-invasive bladder cancer (MIBC), radical cystectomy (RC) is currently the recommended standard treatment. However, RC is associated with a significant risk of perioperative morbidity and mortality, with up to 67% of patients experiencing complications within 90 days of surgery [3, 4]. This reduces the life expectancy of patients and leads to psychological distress [5]. As a result, trimodal therapy (TMT) has emerged as an alternative treatment option for patients who are unable to tolerate RC or who strongly desire to preserve their bladder [6]. The fundamental treatment strategy of TMT entails performing maximum transurethral resection of bladder tumor (TURBT) followed by concurrent administration of chemotherapy and radiation therapy (RT). Briefly, RT and chemotherapy involve a dose of 55–64 Gy delivered over a period of 4–6 weeks in daily fractions of 2–2.75 Gy, targeting the bladder, distal ureter, and proximal urethra (including the prostate), with concurrent 5-fluorouracil plus mitomycin, gemcitabine, or cisplatin. MRI can aid in precise delineation of the TURBT site for radiotherapy planning [7, 8]. The main advantage of TMT is that it preserves bladder function, allowing patients to have a more satisfactory survival experience. However, compared with RC, TMT s ability to control tumors has been questioned. Several articles have recently analyzed the oncological outcomes of TMT and RC, but their results have been conflicting [9–14]. Therefore, it remains unclear whether TMT can provide comparable oncological outcomes to RC.

To evaluate whether TMT can provide comparable oncologic outcomes to RC, we performed this meta-analysis. By using multifactor regression and propensity score matching (PSM), we aimed to reduce the impact of confounding variables and selection bias on the results of the meta-analysis. As a result, the inclusion criteria of our study were limited to those that used these methods, which helped to improve the reliability of our findings.

## Methods

### Search strategy and study selection

Adhering to the guidelines outlined by the Preferred Reporting Items for Systematic Reviews and Meta-Analyses (PRISMA) [15], we conducted an overall thorough selection criteria and statistical analysis of the literature. This study has been registered with the INPLASY registry (registration number: INPLASY202350004). We utilized the following search terms: ("trimodal therapy" OR "radiotherapy" OR "chemoradiotherapy" OR "chemoradiation" OR "bladder-sparing") AND ("radical cystectomy") AND ("bladder cancer" OR "bladder carcinoma") to search for relevant studies in Web of Science, EMBASE, MEDLINE, and the Cochrane Library up to June 2023. To ensure the quality of the included studies, several requirements were satisfied. First, only randomized controlled trials (RCTs) or prospective/retrospective cohort studies were considered. Second, Studies had to compare the effectiveness of trimodal therapy (TMT) and radical cystectomy (RC) in patients diagnosed with muscle-invasive bladder cancer (MIBC). Third, the primary outcome measures had to report overall survival (OS) or cancer-specific survival (CSS). Fourth, multivariable analysis or propensity score matching was required for the survival outcomes analysis. Fifth, letters to the editor, reviews, case-series, and case-reports were not considered. Finally, in the case of multiple studies based on real-world databases, the study providing the most relevant information was selected.

### Data extraction and risk of bias

Our team of two independent reviewers conducted a thorough data extraction process from the relevant studies. The extracted data included study demographics, patient baseline characteristics, and the primary outcome measures of OS and CSS. The reviewers meticulously extracted the data to ensure accuracy and completeness. Study demographics comprised the first author's name, publication year, patient country of origin, study design type, and follow-up duration. Patient baseline characteristics included age at diagnosis, sex distribution, Charlson comorbidity score status, tumor stage classification system used for diagnosis or treatment planning purposes, and tumor grade. To assess the quality of individual studies, the two reviewers evaluated them independently using the Downs and Black tool [16], which ranges from 0 to 28. In cases of disagreement, a third investigator was consulted to resolve them. Additionally, to reduce the risk of duplicate data, we carefully examined the included studies and excluded any overlapping data.

### Statistical analysis

We employed different statistical methods to analyze the data in our study. For continuous and dichotomous baseline variables, we used weighted mean difference (WMD) and risk ratio (RR), respectively, with 95% confidence intervals (CIs) to compare the TMT and RC groups. To compare the OS and CSS outcomes between the two groups, we used hazard ratios (HR) with 95% CIs. We considered a *p*-value of less than 0.05 as statistically significant.

To evaluate the heterogeneity among studies, we used Chi-square-based Q tests and I^2^ statistics. When high heterogeneity was observed, defined as an I^2^ value > 50% and a P value < 0.05, we used a random-effects model (the DerSimonian and Laird method) to determine the pooled effect. If this method was not feasible, we used a fixed-effects method (the Mantel–Haenszel method) for meta-analysis [17]. We assessed possible publication bias by creating Begg's funnel plot and performing Egger's test. Begg's funnel plot evaluates the asymmetry of the distribution of study results and sample sizes, while Egger's test assesses the relationship between the effect size and its standard error. All statistical analyses were performed using Review Manager version 5.4.

## Results

### Study selection and characteristics of studies

**Fig. 1:**
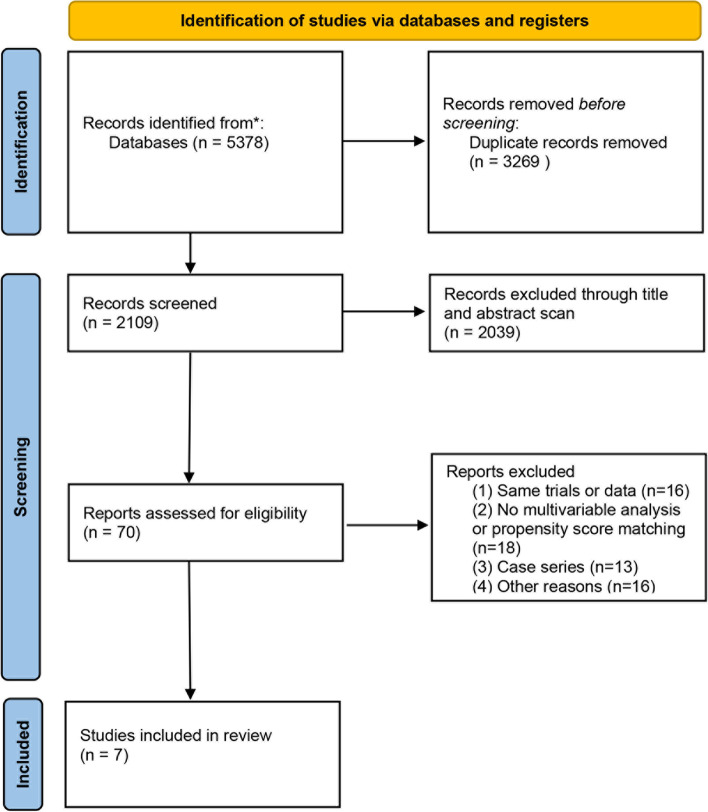
Flow diagram of selection of eligible studies

A flowchart in Fig. 1 illustrates the selection process of relevant literature. Our comprehensive search identified 5378 articles ultimately. We excluded duplicates and studies that didn’t accord with our inclusion criteria based on title, abstract scan, and subsequent full-text review. Ultimately, seven articles with data on 3489 and 13877 patients in the TMT and RC groups, respectively, were included in our meta-analysis [13, 18–23]. Table 1 shows the characteristics and quality assessment of the included studies. All seven articles were retrospective cohort designs. Among them, five articles [18–22] used PSM, in which age, gender, tumor stage, and tumor grade were the most frequent matching factors. All seven articles performed multivariable analysis. Based on the quality ratings, the most of the articles included in our analysis were determined to be of moderate quality.

**Table 1:** Characteristic and quality of retrieved studies

Reference	Year	Country, year	Patient Number (after PSM)		Maximum follow up (years)	PSM	Design	Quality score
			TMT	RC				
Bekelman [23]	2013	SEER, 1995–2005	417	1426	9	None	R	15
Kim [22]	2017	Korea, 2007–2014	32 (29)	308 (50)	5	1,2,5,6,10	R	15
Kulkarni [21]	2017	Single-center, 2008–2013	(56)	(56)	6	1,2,5,6,10	R	16
Williams [20]	2018	SEER, 2002–2011	752 (687)	2448 (687)	10	1,2,4,9,5,7,10	R	19
Zhong [19]	2019	NCDB, 2004–2013	1178 (1002)	7276 (1002)	10	1,5,6,7,8	R	18
Kumar [13]	2021	US Veterans, 2000–2017	163	1472	5	None	R	17
Qiu [18]	2022	CaPRICE, 2005–2014	(891)	(891)	15	1,2,3,4,5,6	R	18

1 = age, 2 = gender, 3 = year at treatment, 4 = education achievement, 5 = clinical T stage, and 6 = Charlson Comorbidity Index, 7 = disease site, 8 = facility type, 9 = marital status, 10 = grade

*R* retrospective, *PSM* Propensity scoring matching

### Baseline characteristics of included patients

Five articles presented individual patient data without propensity score matching, as shown in Supplemental Fig. 1. At baseline, the TMT group exhibited significantly older age (WMD: 6.53 years, 95% CI: 3.91–9.16, *P* < 0.001, I^2^ = 97%), comparable sex distribution (RR: 1.04, 95% CI: 0.96–1.13, *P* = 0.34, I^2^ = 0%), higher rates of comorbidity (RR: 1.56, 95% CI: 1.36–1.80, *P* < 0.001, I^2^ = 0%), lower tumor stage (RR: 0.68, 95% CI: 0.48–0.97, *P* = 0.03, I^2^ = 90%), and lower tumor grade (RR: 0.98, 95% CI: 0.96–0.99, *P* = 0.01, I^2^ = 27%). After propensity score matching, there was no difference between the TMT group and the RC group in terms of age, sex, Charlson comorbidity score, tumor stage, and tumor grade (Supplemental Fig. 2).

### Overall survival

**Fig. 2:**
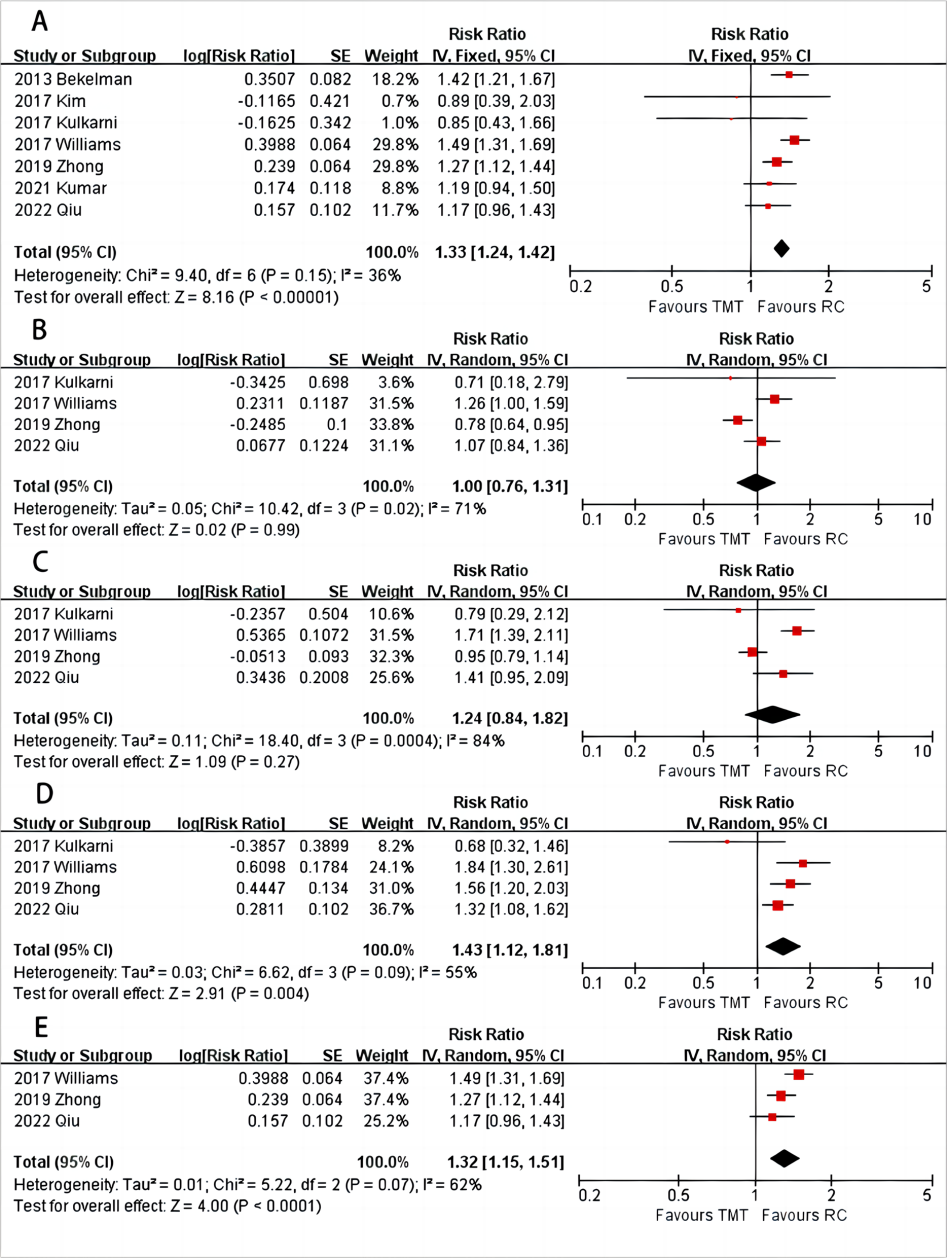
Forest plot of comparisons of overall OS (**A**) and OS at 1-year (**B**), 2-year (**C**), 5-year (**D**), and > 10 year (**E**)

To compare the OS between TMT and RC, the meta-analysis incorporated data from all seven studies. The findings indicated that the RC group had a higher OS compared to the TMT group (Fig. 2A, HR = 1.33, 95%CI: 1.24–1.42, *P* < 0.001, I^2^ = 36%). Further analysis based on the follow-up duration revealed no statistical difference between TMT and RC at 1-year (Fig. 2B, HR = 1, 95%CI: 0.76–1.31, *P* = 0.99, I^2^ = 71%) and 2-year (Fig. 2C, HR = 1.24, 95%CI: 0.84–1.82, *P* = 0.27, I^2^ = 84%). However, patients in the TMT group had inferior OS at 5-year (Fig. 2D, HR = 1.43, 95%CI: 1.12–1.81, *P* = 0.004, I^2^ = 55%) and > 10 year (Fig. 2E, HR = 1.32, 95%CI: 1.15–1.51, *P* < 0.001, I^2^ = 62%).

### Cancer-specific survival

**Fig. 3:**
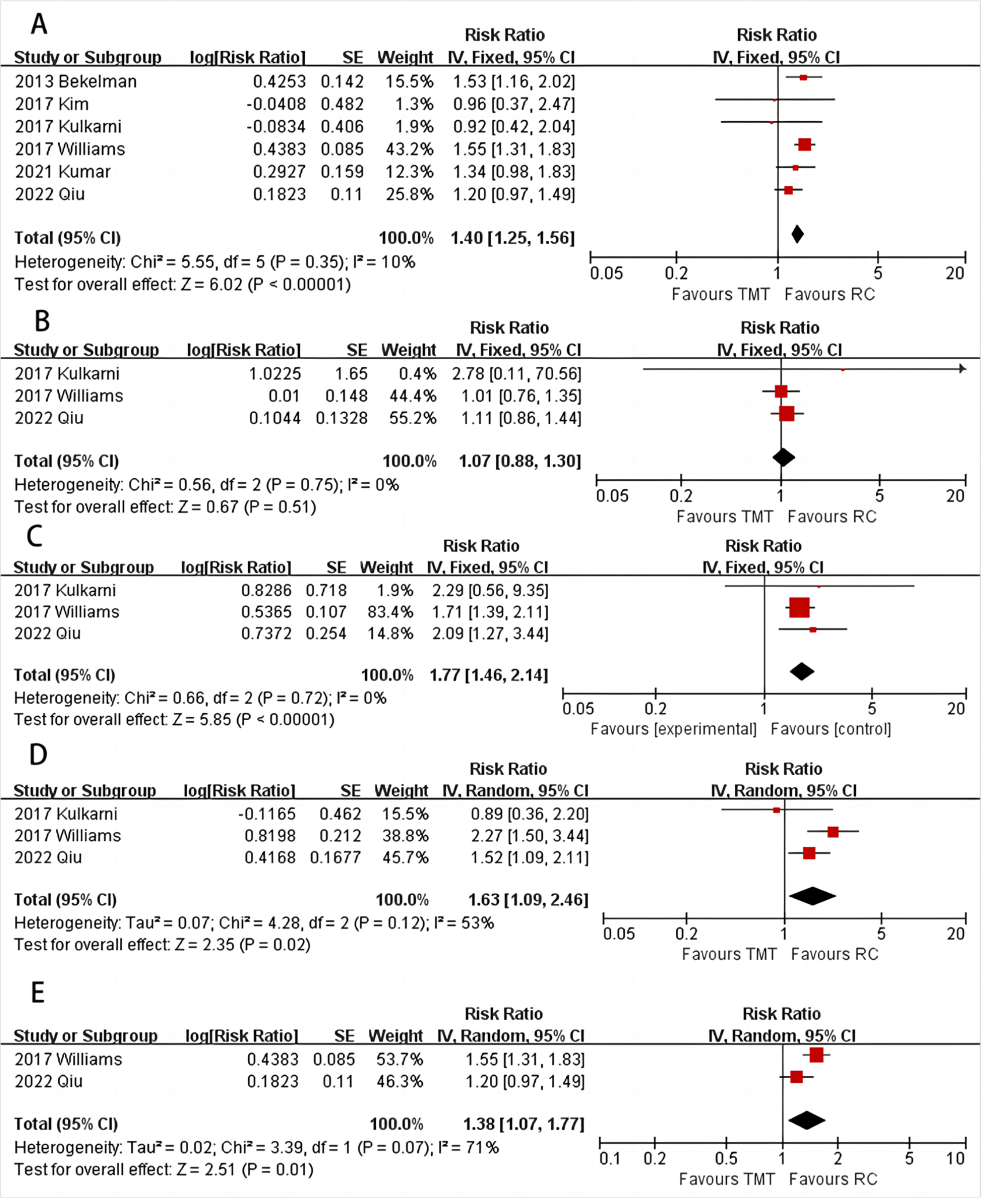
Forest plot of comparisons of overall CSS (**A**) and CSS at 1-year (**B**), 2-year (**C**), 5-year (**D**), and > 10 year (**E**)

All seven studies included in the analysis compared the CSS between TMT and RC. The results showed that patients in the RC group had a higher CSS than those in the TMT group (Fig. 3A, HR = 1.4, 95%CI: 1.25–1.56, *P* < 0.001, I^2^ = 10%). The analysis also showed no significant difference between TMT and RC in terms of 1-year follow-up outcomes (Fig. 3B, HR = 1.07, 95%CI: 0.88–1.3, *P* = 0.51, I^2^ = 0%). However, patients in the TMT group had inferior CSS at 2-year (Fig. 3C, HR = 1.77, 95%CI: 1.46–2.14, *P* < 0.001, I^2^ = 0%), 5-year (Fig. 3D, HR = 1.63, 95%CI: 1.09–2.46, *P* = 0.02, I^2^ = 53%), and > 10-year follow-up (Fig. 3E, HR = 1.38, 95%CI: 1.07–1.77, *P* = 0.01, I^2^ = 71%).

### Publication bias

The Begg's funnel plot of OS (Supplemental Fig. 3) showed no clear evidence of asymmetry, indicating no significant publication bias. This finding was consistent with the p-values obtained from Egger's test for all outcomes, which were greater than 0.05, further supporting the absence of significant publication bias. To minimize the risk of duplicate studies, we carefully reviewed the references of the retrieved articles and only included studies that met our predefined criteria for inclusion.

## Discussion

The objective of this study was to conduct a comprehensive analysis of the conflicting research regarding the oncologic outcomes of RC and TMT, with the aim of providing an accurate evaluation of their disparities. Our findings indicate that, following PSM, there was no statistically significant discrepancy in OS between the RC and TMT groups in the short term (within 2 years). However, the RC group exhibited a significantly higher OS compared to the TMT group at the 5-year and > 10-year time points. Additionally, there was no disparity in CSS between the RC and TMT groups at the 1-year mark. However, from the second year onwards, including the 5-year and > 10-year intervals, the RC group demonstrated a significantly higher CSS compared to the TMT group. Notably, the most significant difference between the two groups was observed at the 2-year mark (*P* < 0.001).

It is noteworthy that a previous meta-analysis (Ding et al. [24]) has evaluated the oncological outcomes of RC and TMT. In their results, TMT had comparable OS and CSS during short-term (1-year, 2-year) and middle-term (5-year) follow-up period, and inferior OS and CSS during long-term follow-up period (> 10 years). However, in our results, TMT had inferior middle-term to long-term OS and inferior short-term to long-term CSS. At baseline, patients undergone TMT appear to be with lower tumor grade and tumor stage. Considering tumor grade and tumor stage are two dominant risk factors of oncological outcomes, the difference of baseline characteristic between two groups may result in bias of final results. Unfortunately, the previous meta-analysis conducted by Ding et al. [24] e extracted several oncological data without PSM or Multivariate regression analysis. To minimize the influence of baseline characteristics between patients undergone TMT and those undergone RC, we only included studies with PSM or Multivariate regression analysis. As can be seen from our results, various characteristics were similar between the two groups after PSM. Apparently, our study has more evidence-based reliability than the previous meta-analysis.

The current selection criteria for TMT rely on clinical and pathological factors; however, a single-institution retrospective study demonstrated that 73% of 212 patients with clinical T2 tumors who underwent RC and did not receive neoadjuvant chemotherapy were found to have pathological T3/T4 or lymph node positivity [25]. This suggests that underestimation of tumor stage before TMT treatment may contribute to the poor long-term prognosis of TMT. In recent years, researchers have explored new biomarkers or molecular markers for patient classification and selection, and immunotherapy is being increasingly used. More and more studies have explored the value of biomarkers in the early diagnosis of bladder cancer. Some proteins in urine such as NMP22, Complement factor H-related protein, BLCA-4, and apolipoproteins [26] as well as various metabolites such as taurine, citrate, succinate [27, 28], desaminotyrosine, erythritol, d-ribose, ribitol, d-fructose, d-mannose, and d-galactose [29] have been found to be biomarkers for the early diagnosis of bladder cancer, and circulating MicroRNA have also been found to be used for the early diagnosis of bladder cancer [30]. Biomarker analysis has suggested that DNA damage response markers, such as MRE11 and ERCC1/2, may predict cancer-specific survival among two independent TMT cohorts [31, 32]. Transcriptome sequencing analysis has also indicated that oncologic outcomes may vary based on different molecular subtypes, and neuroendocrine tumor gene expression signature has been identified in MIBC, which differs from traditional basal/luminal MIBC [33]. In contrast, biomarkers in urine are more specific and less sensitive. Therefore, simultaneous detection of biomarkers in urine and circulation may improve the early detection of bladder cancer. These findings will impact future clinical decisions.

Furthermore, while neoadjuvant chemotherapy has been shown to improve survival in MIBC patients undergoing radical cystectomy, its efficacy in TMT therapy remains uncertain [34, 35]. One study explored the potential of consolidative partial cystectomy and pelvic lymph node dissection to preserve bladder function after TMT treatment and achieved complete remission [36]. Among the patients, 10% had residual tumor invasion and 2% had lymph node metastasis. The 5-year metastasis-free survival, CSS, and OS rates were 97%, 93%, and 91%, respectively. Partial cystectomy did not affect bladder function or quality of life. These findings suggest that consolidation surgery may have a role in TMT therapy.

Our study suggests that in the real world, TMT is mainly used in older patients with relatively early tumor stage and grade [4, 6, 8]. There is no doubt that patients with relatively low-grade and earlier-stage tumors have better tumor prognosis, which is why we emphasize in our study that we must include studies that only do multivariate analysis or PSM, which will make the study results more credible.

Our study also has certain shortcomings. First, all the studies selected by us for inclusion belong to retrospective studies rather than RCT studies, but unfortunately, it is difficult to conduct RCT studies on the comparison of TMT therapy and RC therapy at present. Second, all included studies did not performed subgroup analysis, it is still unclear whether TMT could provide comparable oncological outcomes in specific subgroup patients, and future well- designed studies on this topic is still needed.

In conclusion, our exhaustive investigation and meticulous statistical examination have demonstrated the superiority of RC over TMT in the management of MIBC, particularly with regard to long-term overall survival and specific survival. Consequently, in subsequent clinical treatment decisions, we advocate for the preference of RC over TMT for the treatment of MIBC.

### Supplementary Information

**Additional file 1: Supplemental figure 1.** Forest plot of comparisons of age (A), sex (B), Charlson comorbidity score (C), tumor stage (D) and tumor grade (E) before propensity score matching.

**Additional file 2: Supplemental figure 2.** Forest plot of comparisons of age (A), sex (B), Charlson comorbidity score (C), tumor stage (D) and tumor grade (E) after propensity score matching.

**Additional file 3: Supplemental figure 3.** Begg’s funnel plot of overall OS.

**Additional file 4.** Literature search strategy.

## Data Availability

The original data is available upon reasonable request.

## References

[1] Sung H, Ferlay J, Siegel RL (2021). Global Cancer Statistics 2020: GLOBOCAN Estimates of Incidence and Mortality Worldwide for 36 Cancers in 185 Countries. CA Cancer J Clin.

[2] Xu N, Yao Z, Shang G (2022). Integrated proteogenomic characterization of urothelial carcinoma of the bladder. J Hematol Oncol.

[3] Ghandour R, Singla N, Lotan Y (2019). Treatment Options and Outcomes in Nonmetastatic Muscle Invasive Bladder Cancer. Trends Cancer.

[4] Kamat AM, Hahn NM, Efstathiou JA (2016). Bladder cancer. Lancet.

[5] Lin HY, Ye H, Kernen KM (2018). National Cancer Database Comparison of Radical Cystectomy vs Chemoradiotherapy for Muscle-Invasive Bladder Cancer: Implications of Using Clinical vs Pathologic Staging. Cancer Med.

[6] Ploussard G, Daneshmand S, Efstathiou JA (2014). Critical analysis of bladder sparing with trimodal therapy in muscle-invasive bladder cancer: a systematic review. Eur Urol.

[7] Broughman JR, Vuong W, Mian OY (2020). Current Landscape and Future Directions on Bladder Sparing Approaches to Muscle-Invasive Bladder Cancer. Curr Treat Options Oncol.

[8] Jiang DM, Chung P, Kulkarni GS (2020). Trimodality Therapy for Muscle-Invasive Bladder Cancer: Recent Advances and Unanswered Questions. Curr Oncol Rep.

[9] Cahn DB, Handorf EA, Ghiraldi EM (2017). Contemporary use trends and survival outcomes in patients undergoing radical cystectomy or bladder-preservation therapy for muscle-invasive bladder cancer. Cancer.

[10] Seisen T, Sun M, Lipsitz SR (2017). Comparative Effectiveness of Trimodal Therapy Versus Radical Cystectomy for Localized Muscle-invasive Urothelial Carcinoma of the Bladder. Eur Urol.

[11] Gofrit ON, Nof R, Meirovitz A, et al. Radical cystectomy vs. chemoradiation in T2–4aN0M0 bladder cancer: a case-control study. Urol Oncol. 2015;33(1):19 e11–19 e15. 10.1016/j.urolonc.2014.09.014. 10.1016/j.urolonc.2014.09.014 25445384

[12] Royce T J, Feldman A S, Mossanen M, et al. Comparative Effectiveness of Bladder-preserving Tri-modality Therapy Versus Radical Cystectomy for Muscle-invasive Bladder Cancer. Clin Genitourin Cancer. 2019;17(1):23–31 e23. 10.1016/j.clgc.2018.09.023. 10.1016/j.clgc.2018.09.023 30482661

[13] Kumar A, Cherry DR, Courtney PT (2021). Outcomes for Muscle-invasive Bladder Cancer with Radical Cystectomy or Trimodal Therapy in US Veterans. Eur Urol Open Sci.

[14] Softness K, Kaul S, Fleishman A, et al. Radical cystectomy versus trimodality therapy for muscle-invasive urothelial carcinoma of the bladder. Urol Oncol. 2022;40(6):272 e271–272 e279. 10.1016/j.urolonc.2021.12.015. 10.1016/j.urolonc.2021.12.015 35058142

[15] Page M J, McKenzie J E, Bossuyt P M, et al. The PRISMA 2020 statement: an updated guideline for reporting systematic reviews. BMJ. 2021;372:n71. 10.1136/bmj.n71. 10.1136/bmj.n71 PMC8005924 33782057

[16] Higgins JP, Altman DG, Gøtzsche PC, et al. The cochrane collaboration's tool for assessing risk of bias in randomised trials[J]. Bmj. 2011;343:d5928. 10.1136/bmj.d5928. 10.1136/bmj.d5928 PMC3196245 22008217

[17] DerSimonian R, Laird N (1986). Meta-analysis in clinical trials. Control Clin Trials.

[18] Qiu J, Zhang H, Xu D (2022). Comparing Long-Term Survival Outcomes for Muscle-Invasive Bladder Cancer Patients Who Underwent with Radical Cystectomy and Bladder-Sparing Trimodality Therapy: A Multicentre Cohort Analysis. J Oncol.

[19] Zhong J, Switchenko J, Jegadeesh NK (2019). Comparison of Outcomes in Patients With Muscle-invasive Bladder Cancer Treated With Radical Cystectomy Versus Bladder Preservation. Am J Clin Oncol.

[20] Williams SB, Shan Y, Jazzar U (2018). Comparing Survival Outcomes and Costs Associated With Radical Cystectomy and Trimodal Therapy for Older Adults With Muscle-Invasive Bladder Cancer. JAMA Surg.

[21] Kulkarni GS, Hermanns T, Wei Y, et al. Propensity score analysis of radical cystectomy versus bladder-sparing trimodal therapy in the setting of a multidisciplinary bladder cancer clinic. 2017;35(20):2299-2305. 10.1200/JCO.2016.69.2327 28410011

[22] Kim YJ, Byun S J, Ahn H, et al. Comparison of outcomes between trimodal therapy and radical cystectomy in muscle-invasive bladder cancer: a propensity score matching analysis. 2017;8(40):68996. 10.18632/oncotarget.16576 PMC5620314 28978174

[23] Bekelman JE, Handorf EA, Guzzo T (2013). Radical cystectomy versus bladder-preserving therapy for muscle-invasive urothelial carcinoma: examining confounding and misclassification biasin cancer observational comparative effectiveness research. Value Health.

[24] Ding H, Fan N, Ning Z, et al. Trimodal Therapy vs. Radical Cystectomy for Muscle-Invasive Bladder Cancer: A Meta-Analysis. Front Oncol. 2020;10:564779. 10.3389/fonc.2020.564779. 10.3389/fonc.2020.564779 PMC7591759 33154943

[25] Canter D, Long C, Kutikov A (2011). Clinicopathological outcomes after radical cystectomy for clinical T2 urothelial carcinoma: further evidence to support the use of neoadjuvant chemotherapy. BJU Int.

[26] Oeyen E, Hoekx L, De Wachter S, et al. Bladder Cancer Diagnosis and Follow-Up: The Current Status and Possible Role of Extracellular Vesicles. Int J Mol Sci. 2019;20(4). 10.3390/ijms20040821. 10.3390/ijms20040821 PMC6412916 30769831

[27] Pasikanti KK, Esuvaranathan K, Ho PC (2010). Noninvasive urinary metabonomic diagnosis of human bladder cancer. J Proteome Res.

[28] Pasikanti KK, Esuvaranathan K, Hong Y (2013). Urinary metabotyping of bladder cancer using two-dimensional gas chromatography time-of-flight mass spectrometry. J Proteome Res.

[29] Lin JY, Juo BR, Yeh YH (2021). Putative markers for the detection of early-stage bladder cancer selected by urine metabolomics. BMC Bioinformatics.

[30] Usuba W, Urabe F, Yamamoto Y (2019). Circulating miRNA panels for specific and early detection in bladder cancer. Cancer Sci.

[31] Choudhury A, Nelson LD, Teo MT (2010). MRE11 expression is predictive of cause-specific survival following radical radiotherapy for muscle-invasive bladder cancer. Cancer Res.

[32] Kawashima A, Nakayama M, Kakuta Y (2011). Excision repair cross-complementing group 1 may predict the efficacy of chemoradiation therapy for muscle-invasive bladder cancer. Clin Cancer Res.

[33] Batista da Costa J, Gibb E A, Bivalacqua TJ, et al. Molecular Characterization of Neuroendocrine-like Bladder Cancer. Clin Cancer Res. 2019;25(13):3908–3920. 10.1158/1078-0432.Ccr-18-3558. 10.1158/1078-0432.CCR-18-3558 30952638

[34] Giacalone NJ, Shipley WU, Clayman RH (2017). Long-term Outcomes After Bladder-preserving Tri-modality Therapy for Patients with Muscle-invasive Bladder Cancer: An Updated Analysis of the Massachusetts General Hospital Experience. Eur Urol.

[35] Sherif A, Holmberg L, Rintala E (2004). Neoadjuvant cisplatinum based combination chemotherapy in patients with invasive bladder cancer: a combined analysis of two Nordic studies. Eur Urol.

[36] Horwich A, Babjuk M, Bellmunt J (2019). EAU-ESMO consensus statements on the management of advanced and variant bladder cancer-an international collaborative multi-stakeholder effort: under the auspices of the EAU and ESMO Guidelines Committeesdagger. Ann Oncol.

