# Supplementary material for: Oncological effectiveness of bladder-preserving trimodal therapy versus radical cystectomy for the treatment of muscle-invasive bladder cancer: a system review and meta-analysis
Source: World J Surg Oncol. 2023 Aug 29;21:271. doi: 10.1186/s12957-023-03161-z (PMC10464249; doi:10.1186/s12957-023-03161-z)
Supplement: Supplementary file 4 — Additional file 4. Literature search strategy. [file 12957_2023_3161_MOESM4_ESM.docx]

the literature search strategy of PubMed

#1 search" trimodal therapy "

#2 search" radiotherapy "

#3 search" chemoradiotherapy "

#4 search " chemoradiation"

#5 search " bladder-sparing"

#6 search " radical cystectomy"

#7 search " bladder cancer"

#8 search " bladder carcinoma"

#9 #7 AND #8

#10 #1 OR #2 OR #3 OR #4 OR #5

#11 #6 AND #9 AND #10

the literature search strategy of Embase

#1 search" trimodal therapy "

#2 search" radiotherapy "

#3 search" chemoradiotherapy "

#4 search " chemoradiation"

#5 search " bladder-sparing"

#6 combine using AND #1 OR #2 OR #3 OR #4 OR #5

#7 search " bladder cancer"

#8 search " bladder carcinoma"

#9 combine using AND #7 #8

#10 search " radical cystectomy"

#11 #6 AND #9 AND #10

the literature search strategy of Web of Science

#1 All=" trimodal therapy "

#2 All=" radiotherapy "

#3 All=" chemoradiotherapy "

#4 All= " chemoradiation"

#5 All= " bladder-sparing"

#6 All= " radical cystectomy"

#7 All= " bladder cancer"

#8 All= " bladder carcinoma"

#9 #7 AND #8

#10 #1 OR #2 OR #3 OR #4 OR #5

#11 #6 AND #9 AND #10
